# The optimal dose of intravenous dexamethasone in peripheral nerve blocks: A protocol for systematic review and meta-analysis

**DOI:** 10.1097/MD.0000000000032536

**Published:** 2022-12-30

**Authors:** Yinglong Wu, Yiyong Wei, Donghang Zhang

**Affiliations:** a Department of Anesthesiology, Pu’er People’s Hospital, Pu’er, China; b Department of Anesthesiology, Affiliated Hospital of Zunyi Medical University, Zunyi, China; c Department of Anesthesiology, West China Hospital of Sichuan University, Chengdu, China.

**Keywords:** analgesia, dexamethasone, meta-analysis, peripheral nerve blocks (PNB), randomized controlled trials

## Abstract

Although intravenous dexamethasone prolongs the duration of analgesia for peripheral nerve blocks (PNB), the optimal dose of dexamethasone is not yet determined. This protocol aims to compare the analgesic effects of different doses of intravenous dexamethasone on PNB.

**Methods:**

We will search PubMed, EMBASE, the Cochrane Central register of Controlled Trials (CENTRAL), and Web of Science to identify randomized controlled trials that compared the effects of different doses of intravenous dexamethasone for PNB. The duration of analgesia will be defined as the primary outcome. Secondary outcomes will include pain scores, analgesics consumption >48 hours, and the incidence of adverse effects. RevMan 5.3 software will be used for data analysis.

**Results:**

This study will explore the optimal dose of intravenous dexamethasone for the prolongation of analgesia in PNB.

**Conclusion:**

The results of this study will provide evidence for the dose selection of intravenous dexamethasone in PNB.

## 1. Introduction

Peripheral nerve blocks (PNB) are widely used for multiple types of surgeries due to many beneficial aspects, such as perioperative pain relief, reduced opioids consumption and less opioids-related side effects.^[1,2]^ However, the duration of analgesia provided by single-shot PNB is not long enough resulting from the nature of local anesthetics.^[3,4]^ Dexamethasone, a glucocorticoid with minimal mineralocorticoid effect, has been commonly used to prolong the analgesic duration of PNB, either by perineural or intravenous injection.^[5–7]^ Concerning the “off-label” use and potential neurotoxicity for perineural administration of dexamethasone,^[8–10]^ intravenous route may be more appropriate to use in PNB, because a meta-analysis indicates those 2 routes produce equivalent analgesic effects and show comparable safety profiles.^[5]^ Currently, various dose of intravenous dexamethasone has been suggested to improve the analgesic effects of PNB and several studies compared the effects of different dose of intravenous dexamethasone on PNB,^[11–13]^ but the results were not consistent. Therefore, this systematic review and meta-analysis aims to determine the optimal dose of intravenous dexamethasone for the prolongation of analgesia in PNB.

## 2. Methods

### 2.1. Study registration

This protocol has been registered in the International Prospective Register of Systematic Reviews (registration number: CRD42022378029) and is conducted according to the Preferred Reporting Items for Systematic Evaluation and Meta-Analysis Protocols. Ethical approval is not required for this protocol.

### 2.2. Search strategy

We will search PubMed, CENTRAL, EMBASE, and Web of Science from their inception to November 30, 2022 to identify randomized controlled trials that investigate the analgesic effects of different doses of intravenous dexamethasone for PNB. Language will be restricted to English. The terms used for search will include “dexamethasone,” “nerve block,” and “randomized controlled trials.” We will search additional studies by reviewing the references of relative studies. The detailed search strategy for PubMed is shown in Table 1.

**Table 1:** Search strategy for PubMed.

Number	Search terms
#1	Dexamethasone [MeSH]
#2	Dexamethasone [Title/Abstract]
#3	#1 OR #2
#4	Nerve block [Title/Abstract]
#5	Nerve blockade [Title/Abstract]
#6	Peripheral nerve blockade [Title/Abstract]
#7	Peripheral nerve block [Title/Abstract]
#8	#4 OR #5 OR #6 OR #7
#9	Randomized controlled trial [Title/Abstract]
#10	Clinical trial randomized [Title/Abstract]
#11	Controlled clinical trial [Title/Abstract]
#12	Clinical trial [Title/Abstract]
#13	Randomized [Title/Abstract]
#14	Trial [Title/Abstract]
#15	#9 OR #10 OR #11 OR #12 OR #13 OR #14
#16	#3 AND #8 AND #15

MeSH = medical subject headings.

### 2.3. Inclusion and exclusion

Inclusion criteria: study type: randomized controlled trials; participants: adult patients (>18 years) administered with PNB; comparisons: different doses of intravenous dexamethasone; and primary outcomes: duration of analgesia; secondary outcomes: pain scores, analgesics consumption >48 hours, and the incidence of adverse effects (e.g., nausea and vomiting). Reviews, meta-analysis, retrospective studies, comments, case reports, and conference abstracts will be excluded.

### 2.4. Study selection

**Figure 1.**
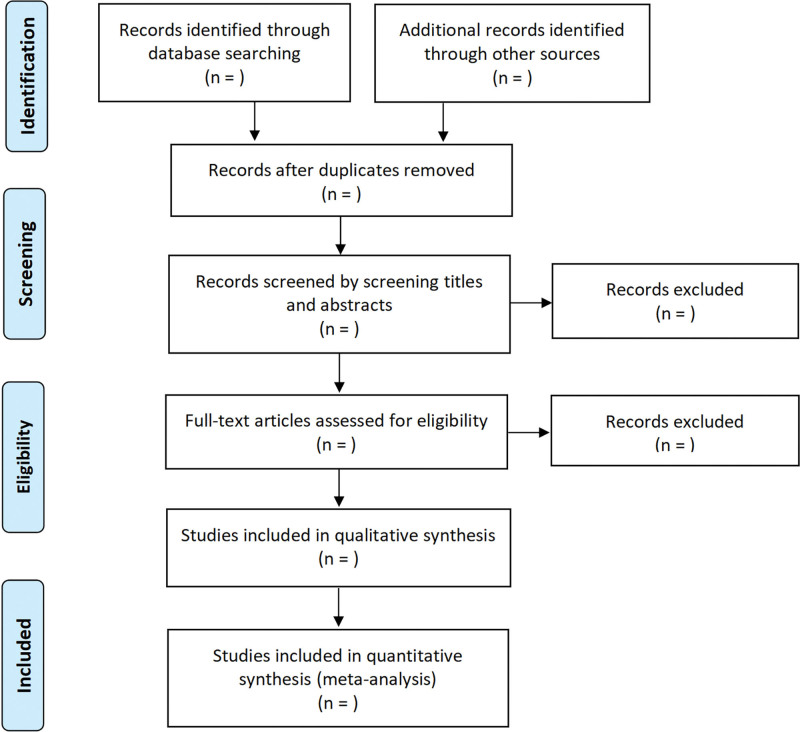
The flow diagram of study selection.

We will identify the eligible studies by screening their title, abstract, followed by reviewing the full-text of potentially relevant articles. Any disagreement will be resolved by discussion. The detailed flowchart for selection is displayed in Figure 1.

### 2.5. Data extraction

We will extract the following information from included studies: publication year, locations, sample size, patients’ characteristics, types of surgery, anesthesia and nerve blocks, interventions and controls, main outcomes, and the strategy of perioperative analgesia. Disagreements will be resolved by discussion.

### 2.6. Risk of bias assessment

We will assess the quality of included studies using the Cochrane Collaboration’s tool,^[14]^ which contains 6 items: random sequence generation (selection bias); allocation concealment (selection bias); blinding of participants and personnel (performance bias); blinding of outcome assessment (detection bias); incomplete outcome data (attrition bias); and selective reporting (reporting bias). The estimated risk of bias for each item will be graded as “low,” “high,” or “unclear.” Disagreements will be discussed with a third author.

### 2.7. Statistical analysis

The mean differences with 95% confidence intervals will be calculated for continuous data, and dichotomous data will be summarized by risk ratios with 95% confidence interval. Statistical heterogeneity will be assessed by the *I*^2^ test. A fixed-effect model will be used to synthesize data when *I*^2^ is <50% (low heterogeneity), otherwise subgroup analysis will be performed to explore the source of heterogeneity, and a random-effect model will be used (significant heterogeneity). Sensitivity analyses will be conducted by excluding 1 study each time to test whether the pooled results are robust. *P* < .05 will be considered statistically significant. The quality of evidence will be judged using the Grading of Recommendations, Assessment, Development and Evaluations approach and rated as “high,” “moderate,” “low,” or “very low.”

## 3. Discussion

Current literature demonstrate that intravenous dexamethasone prolongs the analgesic duration of PNB. However, the doses of dexamethasone used in PNB are various and the dose-response relationship remains unclear. Therefore, this study will compare the effects of different doses of intravenous dexamethasone on PNB, which may provide evidence to guide clinical dose selection of dexamethasone in PNB. Using subgroup analysis, we may further determine the optimal dose of intravenous dexamethasone for specific nerve block or certain local anesthetics.

## Author contributions

**Conceptualization:** Yinglong Wu, Donghang Zhang.

**Data curation:** Yinglong Wu.

**Formal analysis:** Yiyong Wei.

**Methodology:** Yinglong Wu, Yiyong Wei.

**Validation:** Donghang Zhang.

**Writing – original draft:** Yinglong Wu, Donghang Zhang.

**Writing – review & editing:** Yinglong Wu, Donghang Zhang.
